# Lower Healthcare Costs Associated with the Use of a Single-Pill ARV Regimen in the UK, 2004–2008

**DOI:** 10.1371/journal.pone.0047376

**Published:** 2012-10-30

**Authors:** Eduard J. Beck, Sundhiya Mandalia, Roshni Sangha, Mike Youle, Ray Brettle, Mark Gompels, Margaret Johnson, Anton Pozniak, Achim Schwenk, Stephen Taylor, John Walsh, Ed Wilkins, Ian Williams, Brian Gazzard, for the NPMS-HHC Steering Group

**Affiliations:** 1 NPMS-HHC CIC, Coordinating and Analytic Centre, London, United Kingdom; 2 London School of Hygiene & Tropical Medicine, London, United Kingdom; 3 Imperial College, London, United Kingdom; 4 Royal Free Hospital, London, United Kingdom; 5 Edinburgh General Hospital, Edinburgh, United Kingdom; 6 Southmead Hospital, Bristol, United Kingdom; 7 Chelsea and Westminster Hospital, London, United Kingdom; 8 North Middlesex Hospital, London, United Kingdom; 9 Birmingham Heartland Hospital, Birmingham, United Kingdom; 10 St.Mary's Hospital, London, United Kingdom; 11 North Manchester General Hospital, Manchester, United Kingdom; 12 Mortimer Market Centre, London, United Kingdom; Groningen Research Institute of Pharmacy, United States of America

## Abstract

**Aim:**

Investigate the cost and effects of a single-pill versus two- or three pill first-line antiretroviral combinations in reducing viral load, increasing CD4 counts, and first-line failure rate associated with respective regimens at 6 and 12 months.

**Methods:**

Patients on first-line TDF+3TC+EFV, TDF+FTC+EFV, Truvada®+EFV or Atripla® between 1996–2008 were identified and viral load and CD4 counts measured at baseline, six and twelve months respectively. Factors that independently predicted treatment failure at six and twelve months were derived using multivariate Cox's proportional hazard regression analyses. Use and cost of hospital services were calculated at six and twelve months respectively.

**Results:**

All regimens reduced viral load to below the limit of detection and CD4 counts increased to similar levels at six and twelve months for all treatment regimens. No statistically significant differences were observed for rate of treatment failure at six and twelve months. People on Atripla® generated lower healthcare costs for non-AIDS patients at £5,340 (£5,254 to £5,426) per patient-semester and £9,821 (£9,719 to £9,924) per patient-year that was £1,344 (95%CI £1,222 to £1,465) less per patient-semester and £1,954 (95%CI £1,801 to £2,107) less per patient-year compared with Truvada®+EFV; healthcare costs for AIDS patients were similar across all regimens.

**Conclusion:**

The single pill regimen is as effective as the two- and three-pill regimens of the same drugs, but if started as first-line induction therapy there would be a 20% savings on healthcare costs at six and 17% of costs at twelve months compared with Truvada®+EFV, that generated the next lowest costs.

## Introduction

Antiretroviral therapy (ART) has undergone remarkable development since the antiretroviral properties of AZT were first established in 1987 with the subsequent development of dual- and triple-therapy. One of the early problems that people living with HIV (PLHIV) had was the large pill burden associated with triple-therapy when first introduced into routine treatment and care in 1996. The association of lowered adherence with increased pill burden and poorer outcomes has been recognized for some time, in terms of number of pills to be taken *and* the frequency with which they have to be taken [1], [2].

To reduce pill burden, various strategies have been developed over time to produce once-daily dose regimens, combining a number of different drugs into fewer tablets: ‘fixed-dose combinations’ (FDCs). In some FDCs different antiretroviral drugs (ARVs) are combined into one tablet that can be taken once-a-day and improves adherence [3]–[6]. Similar findings have recently been reported with the use of FDCs in the management of hypertension [7].

The production of FDCs precedes their development and use for HIV infection. One of the first FDCs was an oral contraceptive produced in the 1960s followed by the development of maloprim (pyrimethamine+dapsone) and cotrimoxazole (trimethoprim+sulfamethoxazole) in the late 1960's [8]. In 2002 the *WHO Expert Committee on the Use of Essential Drugs* provided the following criteria for FDCs: *“Most essential medicines should be formulated as single compounds. Fixed dose combination products are selected only when the combination has a proven advantage over single compounds administered separately in therapeutic effect, safety, adherence or in delaying the development of drug resistance in malaria, TB and HIV/AIDS.”* [8]. In addition to these criteria identified by the WHO Expert Committee, healthcare costs are now also recognized as an important criterion.

Atripla® is a FDC currently on the market that combines Tenofovir (TDF) with Emtracitabine (FTC) and Efavirenz (EFV). Prior to the introduction of this FDC, TDF was initially prescribed separately with Lamivudine (3TC) and EFV, while after the release of FTC on the market, the three pill regimens also included TDF, FTC and EFV. The next development was a combination of TDF and FTC into one pill – Truvada®; subsequently EFV was added to Truvada® to create Atripla®, a single-pill ARV regimen.

Truvada®+EFV were licensed for first-line induction therapy in 2005 in the UK, while Atripla® was licensed for first-line therapy in the US in 2006 and in 2007 in the UK. Atripla® is currently not licensed for first-line induction therapy in the UK but clinicians can switch from Truvada®+EFV to Atripla® as part of first-line simplification. This particular first-line regimen can therefore be prescribed in four combinations and the aim of this study was to compare the effectiveness and costs of these four combinations in terms of reducing viral load, increasing CD4 counts and treatment failure at 6 and 12 months respectively.

## Methods

The National Prospective Monitoring System on the use, cost and outcome of HIV service provision in UK hospitals - HIV Health-economics Collaboration (NPMS-HHC) has monitored prospectively the *effectiveness*, *efficiency*, *equity* and *acceptability* of treatment and care in participating HIV units since 1996. Using an agreed minimum dataset, standardized data are routinely collected in clinics and transferred to the NPMS-HHC Coordinating and Analytic Centre (CAC) [9]. Since the data were transferred in pseudo-anonymized format, patient consent was not required according to the UK Department of Health in line with international guidelines [10].

### Statistical Analyses

Parametric quantitative data are presented as means with 95% confidence intervals (CIs) or standard deviation (SD) while non-parametric data are presented as medians with inter-quartile range (IQR). Between group comparisons of parametric data with more than two independent groups were tested using one-way-ANOVA while two independent groups were compared using unpaired t-test. Between group comparisons of non-parametric data with more than two independent groups were tested using the Kruskal-Wallis test while two independent groups were compared using the Mann-Whitney U test. Qualitative data by CD4 count strata were tested using the χ^2^ test as well as test for trend and where appropriate these were adjusted using Yates' correction.

### Clinical Outcomes: CD4 counts and viral load when starting ART

Baseline CD4 counts and baseline viral loads were obtained within 4 months before or up to two weeks since starting the first-line regimens under consideration with the closest value to starting the regimen taken as baseline. For 7% of cases baseline CD4 counts and for 10% of cases baseline viral loads could not be obtained and for these patients their baseline CD4 counts or baseline viral loads were imputed using the Multiple Imputations (MI) procedure in SAS. This procedure assumes that the missing baseline CD4 count and baseline viral load data were missing at random (MAR). The missing baseline CD4 counts and baseline viral loads were substituted with an estimated value using a multiple imputation procedure which replaced each missing value with a set of plausible values that represented the uncertainty about the right value to impute [11]. The Markov Chain Monte Carlo (MCMC) method was used to predict mean matching method for imputation, a method that assumed multivariate normality [12]. The MCMC method imputed an observed value that was nearest to the predicted value from the simulated regression model for each missing value imputed.

### Longitudinal changes in CD4 count

Linear mixed models were used to calculate the difference in averages (DAVG) which represent the time weighted difference in CD4 counts from baseline to clinic visits at 6 and 12 months respectively, and where necessary data were transformed to stabilize the variance.

MIXED procedure in SAS was used to fit values of all available CD4 count results since starting first line regimen as a dependent variable. Independent variables included the fixed effects of the treatment groups TDF+3TC+EFV, TDF+FTC+EFV, Truvada®+EFV or Atripla®, clinic visit time points at 6 and 12 months, and treatment groups by study time point interaction. A covariance matrix was used to model the within patient errors. Estimates of change in CD4 count from baseline were obtained from intervention by clinic visit time point interaction. Trends over time are presented as point estimates with 95% confidence intervals (CIs). Multivariable analyses presented were adjusted for other time varying co-variables assumed to have potential confounding or residual effect on the trend of CD4 count changes, including baseline age, sex, ethnic group, clinical status, log10 viral load (VL) and year of starting ART.

### Time to first line treatment failure

Time to first-line treatment failure was estimated from the date of starting the first-line regimen. First-line treatment failure was defined as any change to treatment, this included intensification of regimen by further adding anti-retroviral drug to the regimen or swapping to another anti-retroviral drug class. Simplification of ARVs with no other changes made to the regimen did not constitute treatment failure. Causes of treatment failure included clinical, immunological or virological reasons and others, where adverse effects were the most likely cause [13]. Event time was defined as time from starting first-line treatment until the end of the study periods at 6 or 12 months respectively, or the date of failing first-line TDF+3TC+EFV, TDF+FTC+EFV, Truvada®+EFV or Atripla® before 6 or 12 months respectively. Data were censored either at the end of study period, the date of last clinic visit or date of death if patients had died during the 6 or 12 months study periods. Where patients were on treatments for longer than the study periods at 6 or 12 months, then data were censored at the last date of each respective study periods. Survival curves for overall duration of treatment failure were plotted according to the Kaplan-Meier method and the log-rank method was used to test for differences in survival distributions [14].

Cox's proportional hazards regression models with single variables were initially used to estimate likelihood of treatment failure. All variables found to have a probability of *p*<0.2 in univariable Cox's proportional hazards model were used to build a multivariable model to determine independent predictors of treatment failure while controlling for the other variables in the model. Quantitative data were categorised using median and inter-quartile ranges (IQR), including a separate category for any variables with missing data. This ensured no degrees of freedom were lost when building the multivariable models. The final multivariable models presented were tested for their distributional assumptions using Cox Snell residual plots and adjusted for sex, age, ethnic group, baseline CD4 count and viral load, stage of HIV infection at start of treatment and year of starting ART.

### Use and cost of services

Data on the use of hospital inpatient, outpatient and dayward services between 1^st^ January 2004 and 31^st^ December 2008 were obtained from computerized information systems from 9 UK hospitals participating in this analysis. All patients who started on first line TDF+3TC+EFV, TDF+FTC+EFV, Truvada®+EFV or Atripla® during that period were identified and patients who were known to have transferred from other HIV units were excluded as it was not possible to establish with certainty whether these regimens were indeed their first-line regimen.

The mean number of inpatient days, outpatient visits and dayward visits were calculated for the first six and twelve months on ART respectively. The denominator for those on first-line TDF+3TC+EFV, TDF+FTC+EFV, Truvada®+EFV or Atripla® consisted of the total duration of follow up from when these first-line regimens were started until the end of 6 or 12 months respectively and data were censored as described above. Analyses were stratified by treatment regimens and whether patients had had an AIDS diagnosis or not.

Numerators were calculated by summing the use of inpatient, outpatient or dayward services while on these first-line regimens. Mean use of services and 95% confidence intervals (95%CI) were calculated per patient-semester (6 months) and patient-year (12 months) and summarized by the formula:
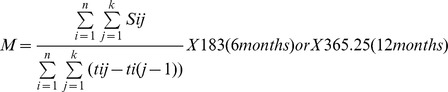
Where

n = total number of individuals;
k = day of censoring;
S_ij_ = use of service of individual i at jth day;
t_ij_ = number of days on first-line TDF+3TC+EFV, TDF+FTC+EFV, Truvada®+EFV or Atripla® for individual i;
M = mean of services at i) 6 months or ii) 12 months

The method used to calculate mean use of services has been employed in a large number of previously published studies [15]–[18]. It is based on a standardized approach that was developed within an European Union Action to analyze HIV healthcare resource utilization across Europe [19], [20] and subsequently adopted by other European Union Concerted Actions [21]. While some would propose assigning mean costs weights to resources used at the individual patient level, this increases the skewing effect of individual outliers and thereby increase the variability of the results. While this may be of interests, the standardized method adopted considered that policy makers are better served with tighter cost-estimates with less of a focus on variability.

The unit cost for an average inpatient day was £511, £101 for an outpatient visit and £413 per dayward visit, unit costs that were obtained from the 2010 NPMS-HHC report [22]. Inpatient, outpatient and dayward costs were obtained by multiplying their means and 95% CIs by their respective unit costs. The costs generated by the use of services were added to the costs of TDF+3TC+EFV, TDF+FTC+EFV, Truvada®+EFV or Atripla®, ‘other’ drugs, and tests and procedures performed. Separate analyses will be presented for those PLHIV who had developed AIDS and those who had not (non-AIDS). The costs for the different ART regimens were prices negotiated by the London HIV Consortium in 2008 with pharmaceutical companies. During 2008 London ARV prices were on average 9% below out-of-London prices, but the latter have since decreased to come in line with London prices (Peter Sharott, personal communication 2011). Furthermore, the costs for ‘other’ drugs, tests and procedures were weighted by stage of HIV infection: non-AIDS or AIDS. The study was performed from a public service perspective [15] and costs for, ‘other’ drugs, tests and procedures performed, were also obtained from the 2010 NPMS-HHC report [22]. Costs were calculated in UK pounds (2008 prices) but not discounted given the short study periods of six and twelve months respectively. All analyses were performed using SAS version 9.1.3 statistical software and all significance tests presented are two-tailed.

## Results

The total number of patients who started on the four regimens was 1,448, of whom 25% had been diagnosed with AIDS (Table 1). Among both groups of patients, the largest proportion had been started on Atripla®, followed by Truvada®+Efavirenz, TDF+3TC+EFV and least number of patients started on TDF+FTC+EFV.

**Table 1:** Demographic characteristics and baseline viral load and CD4 count for all patients on first-line regimens.

	Total = 1448									
	Non-AIDS					AIDS				
	n = 1122					n = 326				
	Atripla®	Truvada®+EFV	TDF+FTC+EFV	TDF+3TC+EFV	*p-value*	Atripla®	Truvada®+EFV	TDF+FTC+EFV	TDF+3TC+EFV	
	N = 681 (61%)	N = 248 (22%)	N = 64 (6%)	N = 129 (15%)		N = 168 (52%)	N = 78 (24%)	N = 18 (6%)	N = 62 (19%)	
Sex										
Female	96 (14.1)	29 (11.7)	13 (20.3)	16 (12.4)	*0.323*	31 (18.5)	12 (15.4)	1 (5.6)	7 (11.3)	*0.350*
Male	585 (85.9)	219 (88.3)	51 (79.7)	113 (87.6)		137(81.6)	66 (84.6)	17 (94.4)	55 (88.7)	
Mean (SD) age start										
ART (years)	37.1 (8.7)	37.7 (8.6)	37.5 (9.2)	38.0 (9.4)	*0.617*	40.6 (9.9)	41.0 10.8)	39.8 (10.1)	39.2 (10.0)	*0.724*
Ethnic group										
Not available	43 (6.3)	8 (3.2)	4 (6.3)	13 (10.1)	*0.120*	18 (10.7)	2 (2.6)	0 (0.0)	13 (21.0)	*0.026*
Other	104 (15.3)	33 (13.3)	5 (7.8)	12 (9.3)		18 (10.7)	6 (7.7)	3 (16.7)	6 (9.7)	
Black African	97 (14.2)	41 (16.5)	13 (20.3)	23 (17.8)		32 (19.1)	13 (16.7)	2 (11.1)	12 (19.4)	
Caucasian	437 (64.2)	166 (66.9)	42 (65.6)	81 (62.8)		100 (59.5)	57 (73.1)	13 (72.2)	31 (50.0)	
Median (IQR)	82707	135683	169918	147506	*<0.001*	97219	228339	265726	256268	*<0.001*
Baseline viral load copies/ml	(28206 to 231921)	(66954 to 355190)	(54029 to 373252)	(46400 to 459532)		(34636 to 328535)	(100000 to 500000)	(103586 to 445000)	(98300 to 500000)	
Mean (SD) baseline	293	242	254	238	*<0.001*	252	245	202	194	
CD4 T-cell count cell/mm^3^	(201 to 382)	(152 to 341)	(161 to 363)	(135 to 337)		(137 to 359)	(123 to 327)	(153 to 241)	(122 to 260)	*0.050*

No significant differences were observed in terms of age when starting first-line ART, sex and past or current history of injecting drugs for patients starting on the different regimens. Some minor differences were noted among the ethnic background of AIDS patients (Table 1).

### Viral load and CD4 count at 6 and 12 months induction ART

**Figure 1:**
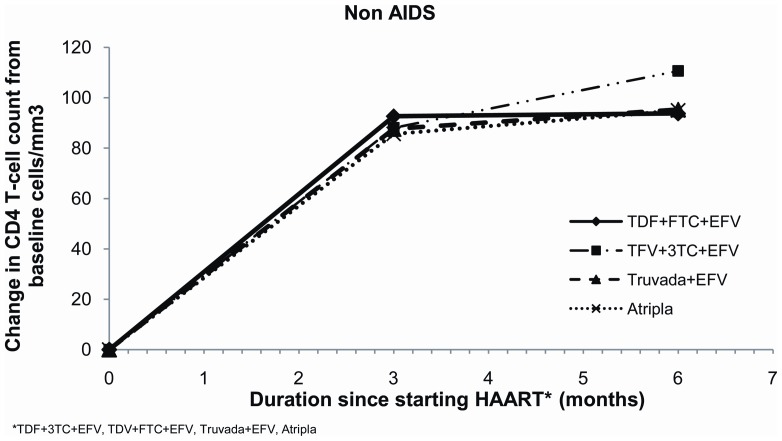
Change in CD4 count from baseline for non-AIDS patients at 6 months for the four treatment regimens.

**Figure 2:**
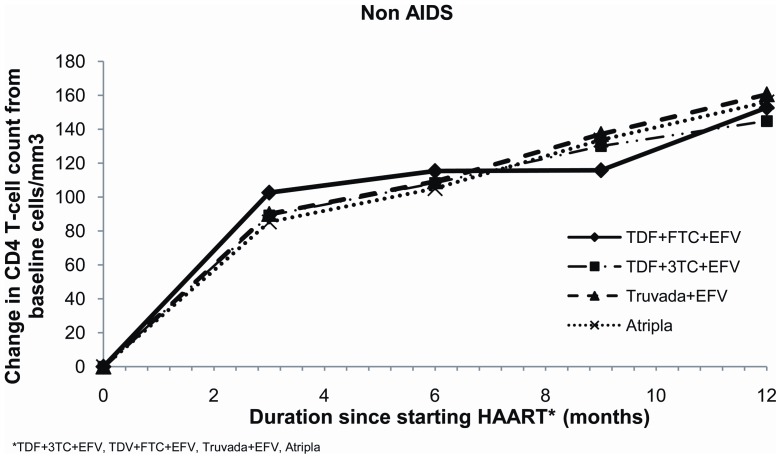
Change in CD4 count from baseline for non-AIDS patients at 12 months for the four treatment regimens.

Baseline viral loads for non-AIDS patients on Atripla® were significantly lower than for those patients on the other regimens (Table 1). However the median VL for all non-AIDS patients had become undetectable at 6 months at less than 50 copies/ml (IQR <50 and <50 copies/ml) and this was still the case at 12 months. Mean CD4 count for all non-AIDS patients starting ART was <350 cell/mm3, with baseline CD4 count for patients on Atripla® significantly higher than that for other regimens; baseline CD4 counts for the other three regimens were similar (Table 1). Increases in mean CD4 count at 6 months were, however, similar across all four regimens (Table 2; Figure 1) as were mean increases at 12 months (Table 2; Figure 2).

**Table 2:** CD4 T-cell count changes at 6 and 12months for non-AIDS and AIDS patients on different first-line regimens.

	Mean (95% CI) increase in CD4 T-cell count from baseline cells/mm^3^							
	Atripla®		Truvada®+EFV		TDF+FTC+EFV		TDF+3TC+EFV	
	6 months	12 months	6 months	12 months	6 months	12 months	6 months	12 months
**Total = 1122**	**N = 681**		**N = 248**		**N = 64**		**N = 129**	
Non-AIDS	95 (82 to 108)	156 (138 to 174)	95 (74 to 117)	161 (134 to 187)	94 (46 to 142)	153 (102 to 203)	111 (82 to 139)	145 (109 to 181)
**Total = 326**	**N = 168**		**N = 78**		**N = 18**		**N = 62**	
AIDS	105 (82 to 128)	158 (124 to 191)	87 (53 to 122)	171 (128 to 213)	105 (1 to 215)	188 (1 to 407)	104 (59 to 149)	145 (89 to 201)

**Figure 3:**
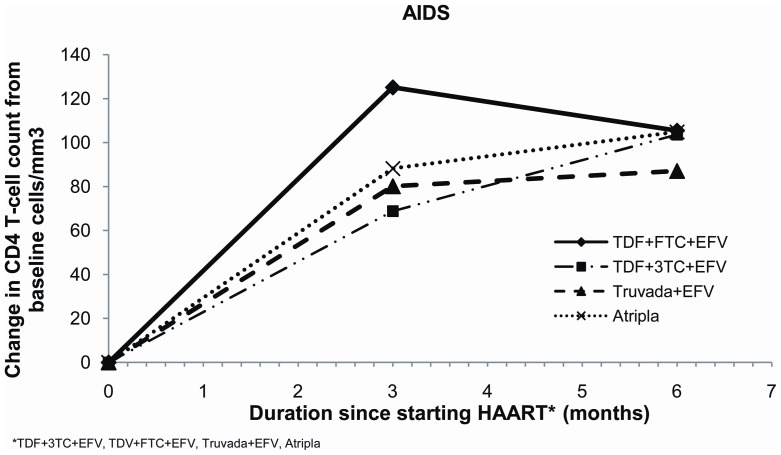
Change in CD4 count from baseline for AIDS patients at 6 months for the four treatment regimens.

**Figure 4:**
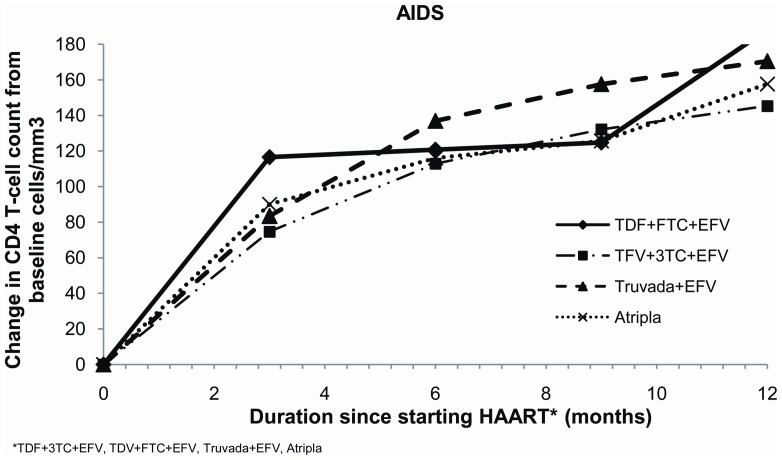
Change in CD4 count from baseline for AIDS patients at 12 months for the four treatment regimens.

Baseline viral loads for AIDS patients on Atripla® were significantly lower than for those patients on other regimens (Table 1). However, median viral load for all AIDS patients at 6 months had become undetectable at less than 50 copies/ml (IQR <50 and <50 copies/ml) and remained undetectable at 12 months. Mean baseline CD4 count for AIDS patients were all less than 250 cells/mm3, with TDF+3TC+EFV patients having the lowest CD4 count (Table 1); increases in CD4 counts were similar for all AIDS patients at 6 (Table 2; Figure 3) and 12 months after starting all ART regimen (Table 2; Figure 4).

### Treatment Failure at 6 and 12 months

No significant statistical differences were observed between regimens in terms of likelihood of treatment failure at 6 and 12 months (Tables 3 and 4).

**Table 3:** Multivariable Cox's proportional hazards regression model showing likelihood of first line treatment failure for the four treatment combinations at 6 months.

Variable	Total started first line HAART		Rx failure at 6 months	Hazard Ratio*	95% CI	p-value
	*N = 1448*		n = 176 (%)			
ART at	TDF+FTC+EFV	82	10 (12.2%)	0.97	(0.37 to 2.54)	0.945
start of 1^st^	TDF+3TC+EFV	191	32 (16.8%)	0.74	(0.24 to 2.30)	0.608
line	Truvada**®**+EFV	326	35 (10.7%)	0.74	(0.33 to 1.65)	0.459
HAART	Atripla**®**	849	99 (11.7%)	1		

* Adjusted for sex, age, ethnic group, baseline CD4 count, baseline viral load, stage of HIV at start of ART and year of starting first-line ART.

**Table 4:** Multivariable Cox's proportional hazards regression model showing likelihood of first line treatment failure for the four treatment combinations at 12 months.

Variable	Total started first line HAART		Rx failure at 12 months	Hazard Ratio*	95% CI	p-value
	*N = 1448*		n = 261 (%)			
ART at	TDF+FTC+EFV	82	16 (19.5%)	0.98	(0.39 to 2.45)	0.963
start of 1^st^	TDF+3TC+EFV	191	53 (27.8%)	1.41	(0.64 to 3.11)	0.389
line	Truvada**®**+EFV	326	56 (17.2%)	0.94	(0.48 to 1.85)	0.855
HAART	Atripla**®**	849	136 (16.0%)	1		

* Adjusted for sex, age, ethnic group, baseline CD4 count, baseline viral load, stage of HIV at start of ART and year of starting first-line ART.

### Use and cost of services

For non-AIDS patients, the cost of use of services at 6 months ranged from £5,340 (95%CI £5,254 to £5,426) for those on Atripla® to £7,554 (95%CI £7,243 to £7,864) for those on TDF+3TC+EFV. Patients on Atripla® generated the lowest cost, as their use of inpatient services was significantly lower than that generated by patients on the other regimens. For Truvada®+EFV the cost of services at 6 months was £6,684 (95%CI £6,476 to £6,891) which was £1,344 (95%CI £1,222 to £1,465) per patient-semester more expensive compared with Atripla® (Table 5).

**Table 5:** Use and cost of hospital services for non-AIDS patients at 6 and 12 months respectively for the four treatment regimens.

	Non-AIDS							
	N = 1122							
	6 months	12 months	6 months	12 months	6 months	12 months	6 months	12 months
	Atripla®		Truvada®+EFV		TDF+FTC+EFV		TDF+3TC+EFV	
	N = 681		N = 248		N = 64		N = 129	
Mean IP days	0.71	0.84	3.00	3.51	3.51	4.27	3.83	5.31
(95% CI)	(0.65 to 0.78)	(0.77 to 0.91)	(2.78 to 3.22)	(3.26 to 3.76)	(3.04 to 3.97)	(3.74 to 4.81)	(3.48 to 4.18)	(4.88 to 5.74)
Mean OP visits	6.53	9.86	6.84	11.31	5.80	9.70	6.15	10.05
(95% CI)	(6.34 to 6.73)	(9.61 to 10.11)	(6.51 to 7.17)	(10.87 to 11.75)	(5.20 to 6.40)	(8.90 to 10.49)	(5.71 to 6.59)	(9.46 to 10.63)
Mean DW visits	1.00	1.42	1.34	2.49	1.15	1.85	1.38	2.25
(95% CI)	(0.92 to 1.07)	(1.33 to 1.52)	(1.19 to 1.49)	(2.28 to 2.70)	(0.88 to 1.42)	(1.50 to 2.20)	(1.17 to 1.59)	(1.97 to 2.53)
IP costs	£364	£429	£1,533	£1,794	£1,791	£2,184	£1,956	£2,714
(95% CI)	(£331 to £398)	(£391 to £467)	(£1,420 to £1,646)	(£1,668 to £1,920)	(£1,552 to £2,031)	(£1,912 to £2,456)	(£1,777 to £2,134)	(£2,495 to £2,932)
OP costs	£660	£996	£691	£1,142	£586	£980	£621	£1,015
(95% CI)	(£640 to £680)	(£971 to £1,021)	(£658 to £725)	(£1,098 to £1,186)	(£525 to £646)	(£899 to £1,060)	(£577 to £666)	(£956 to £1,074)
DW costs	£412	£587	£555	£1,029	£473	£764	£571	£928
(95% CI)	(£379 to £444)	(£548 to £627)	(£493 to £616)	(£943 to £1,115)	(£362 to £585)	(£619 to £909)	(£484 to £659)	(£812 to £1,043)
Cost of ART	£3,208	£6,416	£3,208	£6,417	£3,661	£7,323	£3,709	£7,417
Cost of non-ART drugs	£499	£998	£499	£998	£499	£998	£499	£998
Cost of tests and procedures	£198	£396	£198	£396	£198	£396	£198	£396
Total costs	**£5,340**	**£9,821**	**£6,684**	**£11,775**	**£7,209**	**£12,643**	**£7,554**	**£13,467**
(95% CI)	(£5,254 to £5,426)	(£9,719 to £9,924)	(£6,476 to £6,891)	(£11,520 to 12,031)	(£6,797 to £7,620)	(£12,146to £13,141)	(£7,243 to £7,864)	(£13,075 to £13,860)

By twelve months, annual cost per patient-year for non-AIDS patients ranged from £9,821 (95%CI £9,719 to £9,924) for patients on Atripla® to £13,467 (95%CI £13,075 to £13,860) per patient-year for patients on TDF+3TC+EFV. For Truvada®+EFV the cost of services at 12 months was £11,775 (95%CI £11,520 to £12,031) which was £1,954 (95%CI £1,801 to £2,107) per patient-year more expensive compared with Atripla® (Table 5). Again the main difference was the reduced use of inpatient services of those on Atripla® compared with the other regimens.

For AIDS patients at 6 months, cost of services ranged from £9,123 (95%CI £8,782 to £9,465) for TDF+FTC+EFV to £15,061 (95%CI £14,360 to £15,762) for those on TDF+3TC+EFV. The cost at 6 months for Atripla® was £10,836 (95%CI £10,563 to £11,110) and the difference with Truvada®+EFV was only £489 (95% £321 to £655) per semester (Table 6).

**Table 6:** Use and cost of services for AIDS patients at 6 and 12 months respectively for the four treatment regimens.

	AIDS							
	N = 326							
	6 months	12 months	6 months	12 months	6 months	12 months	6 months	12 months
	Atripla®		Truvada®+EFV		TDF+FTC+EFV		TDF+3TC+EFV	
	N = 168		N = 78		N = 18		N = 62	
Mean IP days	3.3	4.6	3.1	3.3	0.0	0.0	9.1	12.2
(95% CI)	(3.0 to 3.6)	(4.3 to 5.0)	(2.7 to 3.5)	(2.8 to 3.7)	(0.0 to 0.0)	(0.0 to 0.0)	(8.3 to 9.9)	(11.2 to 13.2)
Mean OP visits	7.3	11.1	8.0	12.8	5.5	8.9	7.8	12.8
(95% CI)	(6.9 to 7.8)	(10.5 to 11.6)	(7.4 to 8.67)	(12.0 to 13.7)	(4.3 to 6.7)	(7.3 to 10.4)	(7.1 to 8.6)	(11.8 to 13.8)
Mean DW	1.9	2.9	3.2	5.0	1.2	1.6	3.6	5.0
visits	(1.7 to	(2.6 to	(2.80 to	(4.4 to	(0.6 to	(0.9 to	(3.1 to	(4.3 to
(95% CI)	2.1)	3.2)	3.62)	5.5)	1.7)	2.2)	4.1)	5.6)
IP costs	£1,693	£2,369	£1,561	£1,661	£0	£0	£4,661	£6,214
(95% CI)	(£1,549 to £1,837)	(£2,196 to £2,542)	(£1,356 to £1,767)	(£1,444 to £1,879)	(£0 to £0)	(£0 to £0)	(£4,249 to £5,074)	(£5,702 to £6,725)
OP costs	£741	£1,117	£811	£1,295	£556	£895	£793	£1,293
(95% CI)	(£699 to £783)	(£1,065 to £1,169)	(£746 to £876)	(£1,211 to £1,380)	(£439 to £673)	(£738 to £1,051)	(£717 to £869)	(£1,189 to £1,397)
DW costs	£776	£1,192	£1,325	£2,050	£487	£654	£1,479	£2,049
(95% CI)	(£688 to £863)	(£1,082 to £1,303)	(£1,155 to £1,495)	(£1,833 to £2,267)	(£263 to £711)	(£381 to £927)	(£1,267 to £1,692)	(£1,782 to £2,316)
Cost of ART	£3,208	£6,416	£3,208	£6,417	£3,661	£7,323	£3,709	£7,417
Cost of non-ART drugs	£3,664	£7,327	£3,664	£7,327	£3,664	£7,327	£3,664	£7,327
Cost of tests and procedures	£756	£1,511	£756	£1,511	£756	£1,511	£756	£1,511
Total costs	**£10,836**	**£19,933**	**£11,325**	**£20,261**	**£9,123**	**£17,710**	**£15,061**	**£25,811**
(95% CI)	(£10,563 to £11,110)	(£19,597 to 20,268)	(£10,884 to £11,765)	(£19,743 to £20,780)	(£8,782 to £9,465)	(£17,281 to £18,139)	(£14,360 to £15,762)	(£24,930 to £26,692)

At 12 months, the cost per AIDS patient-year ranged from £17,710 (95% CI £17,281 to £18,139) for those on TDF+FTC+EFV to £25,811 (95% CI £24,930 to £26,692) per patient-year for patients on TDF+3TC+EFV. The annual cost on Atripla® was £19,933 (95% CI £19,597 to £20,268) and differed by £328 (95% CI 146 to 512) per patient-year with the Truvada®+EFV regimen (Table 6).

## Discussion

All regimens displayed similar effectiveness in terms of reducing viral load to undetectable serum levels by 6 months and maintained that at 12 months; all regimens increased CD4 count to similar levels by 6 and 12 months and no statistically significant differences were observed in terms of treatment failure rates at 6 months and 12. These outcome were achieved, despite that median VL was lower and mean baseline CD4 counts for non- AIDS patients on Atripla were higher than for those on other regimens. While the number of patients failing their regimen was similar across all regimens, those on Atripla used fewer inpatient or dayward services. The observed percentage of patients failing Atripla within one year was 16% and this was similar to the 20% recently reported from a single London centre [23]. Authors from that study indicated that most patients had stopped Atripla® due to central nervous system adverse events.

In term of use and cost of services for non-AIDS patients, Atripla® generated the lowest healthcare costs at six and twelve months whereas six monthly or annual costs for AIDS patients were similar across all regimens. Starting with higher CD4 counts, not using inpatient services, even for those who failed, and using fewer dayward services, all contributed to lower healthcare costs. The lowest costs among AIDS patients were generated by patient on TDF+FTC+EFV. However, as there were only 18 patients on this regimen - none of whom used inpatient services – questions can be raised concerning the representative nature of this and the other results observed among the AIDS patients. Furthermore waiting until a person living with HIV develops an AIDS defining condition before starting them on ART would be inappropriate; one wants to diagnose people living with HIV early and review them regularly in a controlled clinical situation and start ART well before they develop AIDS [24]. While some guidelines still recommend starting ART when CD4 count ≤350 cells/mm3 [25], recent US guidelines recommend starting when CD4 ≤500 cells/mm3, while some US clinicians recommend to start ART when people are diagnosed with HIV irrespective of CD4 count [26].

The analyses as presented have their limitations. Firstly, some of the comparator groups had small number of patients many of whom were seen in London clinics. Secondly first viral load or CD4 count when starting these regimens could not be retrieved for a small number of subjects and these had to have their viral load and CD4 count imputed. Thirdly, the data available for operational research are by definition observational data [27] and while the analyses were stratified for potential confounders, some residual confounding may have remained and affected the results. However, despite the inherent potential problem associated with observational data, if one wants to analyze ‘real-life’ service provision or programmes, by necessity one has to rely on observational data [27].

The report of a 2003 WHO meeting on *Fixed-Dose Combinations for HIV/AIDS, Tuberculosis, and Malaria* not only provided evidence of the effectiveness of generic FDCs against HIV but also tried to place the role of FDCs within the broader context of diverse pharmacological interventions [8]. A number of studies have demonstrated the effectiveness of FDCs in resource limited situations [28]–[29] partly through the improved adherence observed with a single pill [30]. The improvement in adherence subsequent to the use of a single pill regimen has also been demonstrated in high-income countries [31]–[33]. Two recent US studies demonstrated that the optimum use of single pill regimens also lowered healthcare costs [32], [33] and a recent Italian study demonstrated the cost-effectiveness of a single pill regimen [34].

Optimizing drug regimens is one of the five pillars of WHO and UNAIDS' Treatment 2.0 policy, and includes *“reducing pill burden by developing ‘one pill a day’ (or less often) fixed-dose combinations (FDCs)”* and its 2020 Goal is the availability of *“effective, affordable, one pill, once-daily potent ARV regimens with minimal toxicities or drug interactions and high barriers to resistance are available in lower and middle-income countries”* [35]. Apart from improving long-term adherence, especially as the number of older people living with HIV are increasing, the use of such FDC may also reduce the cost of treatment and care in these resource-limited countries though this will have to be demonstrated [36], [37].

During times when Governments in high-income countries are also cutting health and welfare budgets to ameliorate rising healthcare costs [38], healthcare interventions need to be assessed in terms of the *cost* of the regimen as well as their *effectiveness*. If it is considered to be appropriate to start a person living with HIV on a regimen of TDF, FTC and EFV, strong clinical and financial arguments can be made for starting this person on single-pill Atripla® as first-line therapy. While Atripla® is currently not licensed for induction therapy in the UK, a substantial number of UK clinicians have started using Atripla® as first-line induction therapy. If Atripla® is started as induction therapy, this avoids the extra costs associated with first starting patients with Truvada®+EFV as induction therapy and only switching to Atripla® after 6 months as part of treatment simplification.
